# Effect of EEG Referencing Methods on Auditory Mismatch Negativity

**DOI:** 10.3389/fnins.2017.00560

**Published:** 2017-10-10

**Authors:** Yatin Mahajan, Varghese Peter, Mridula Sharma

**Affiliations:** ^1^The MARCS Institute for Brain, Behaviour and Development, Western Sydney University, Penrith, NSW, Australia; ^2^The HEARing CRC, Melbourne, VIC, Australia; ^3^Department of Linguistics, Australian Hearing Hub, Macquarie University, Sydney, NSW, Australia

**Keywords:** mismatch negativity, event related potential, reference, REST

## Abstract

Auditory event-related potentials (ERPs) have consistently been used in the investigation of auditory and cognitive processing in the research and clinical laboratories. There is currently no consensus on the choice of appropriate reference for auditory ERPs. The most commonly used references in auditory ERP research are the mathematically linked-mastoids (LM) and average referencing (AVG). Since LM and AVG referencing procedures do not solve the issue of electrically-neutral reference, Reference Electrode Standardization Technique (REST) was developed to create a neutral reference for EEG recordings. The aim of the current research is to compare the influence of the reference on amplitude and latency of auditory mismatch negativity (MMN) as a function of magnitude of frequency deviance across three commonly used electrode montages (16, 32, and 64-channel) using REST, LM, and AVG reference procedures. The current study was designed to determine if the three reference methods capture the variation in amplitude and latency of MMN with the deviance magnitude. We recorded MMN from 12 normal hearing young adults in an auditory oddball paradigm with 1,000 Hz pure tone as standard and 1,030, 1,100, and 1,200 Hz as small, medium and large frequency deviants, respectively. The EEG data recorded to these sounds was re-referenced using REST, LM, and AVG methods across 16-, 32-, and 64-channel EEG electrode montages. Results revealed that while the latency of MMN decreased with increment in frequency of deviant sounds, no effect of frequency deviance was present for amplitude of MMN. There was no effect of referencing procedure on the experimental effect tested. The amplitude of MMN was largest when the ERP was computed using LM referencing and the REST referencing produced the largest amplitude of MMN for 64-channel montage. There was no effect of electrode-montage on AVG referencing induced ERPs. Contrary to our predictions, the results suggest that the auditory MMN elicited as a function of increments in frequency deviance does not depend on the choice of referencing procedure. The results also suggest that auditory ERPs generated using REST referencing is contingent on the electrode arrays more than the AVG referencing.

## Introduction

Event-related potentials (ERPs) are readily used to assess the brain function in response to sensory events. Excellent temporal resolution (in the order of milliseconds) and cost-effectiveness are two major advantages of the ERPs compared to other neuroimaging procedures. The high temporal resolution ability of the ERPs have been utilized to investigate, low-level cognitive functions such as encoding of sounds (Ponton et al., 2000; Mahajan and McArthur, 2012; Gilley et al., 2016), high-level functions such as attention, working memory, and language (Hillyard et al., 1973; SanMiguel et al., 2008; Peter et al., 2014; Mandikal Vasuki et al., 2017) and the functions that fall in between low and high cognitive functions such as auditory memory, sound discrimination, involuntary attention (Escera et al., 2000; Schröger et al., 2000; Winkler, 2007). Researchers have also been using basic auditory ERPs to assess and to identify auditory perceptual processing abilities and disabilities in children, adolescents, and older adults (Wible et al., 2002; Bishop et al., 2007; McArthur et al., 2009). Auditory ERPs are used to validate and document changes in auditory processing ability after intervention, and to assess auditory plasticity of the brain after hearing rehabilitation (Sharma et al., 2002, 2005, 2014; McArthur et al., 2009).

Despite its usefulness in various fields and populations, the recording of reliable EEG is dependent heavily on technical EEG recording-related factors. The technical issue of “choice of EEG referencing” has been a matter of debate for years (Kayser and Tenke, 2010). There has been no common consensus yet on the choice of appropriate referencing procedure in the ERP research laboratories across the world (Kayser and Tenke, 2010; Luck, 2014; Chella et al., 2016). The process of referencing in ERP recordings is essential as the electric potential measured at a particular electrode on the scalp is relative to the activity of a reference electrode placed elsewhere either on the scalp or non-cephalic placements; i.e., the electrical activity measured at a particular electrode is actually the potential difference between EEG signal measured at electrode and the reference cite. Selecting an appropriate reference for EEG recording becomes a crucial process as the choice of reference can induce changes in the EEG recording and subsequently the ERP analyses i.e., latency, magnitude, and spatial changes in the ERPs (Kayser and Tenke, 2010; Tian and Yao, 2013). For a more detailed technical description of referencing procedure, see Luck (2014).

Theoretically, the choice of reference location for EEG recordings should be electrically neutral or in other words, the referencing procedure should result in a neutral potential (Yao, 2001; Kayser and Tenke, 2010; Qin et al., 2010). However, a neutral position on the human body doesn't exist (Yao, 2001; Nunez and Srinivasan, 2006) and any reference position on the body would introduce an electric potential of its own. This may result in compromised quality of the recorded EEG and in turn the final ERPs. Many referencing positions that are subjectively considered relatively neutral such as the vertex (Pang and Taylor, 2000; Tonnquist-Uhlen et al., 2003), the nose (Alho and Sinervo, 1997; Sussman et al., 2008), the ear lobes (Takeshita et al., 2002), nape of the neck (Katznelson, 1981), and the linked mastoids (Peter et al., 2010; Mahajan and McArthur, 2013, 2015) have been used to quantify auditory ERPs. Widely used linked mastoid (LM) referencing procedure (especially when using auditory ERPs) that represent offline mathematically linking the right and left mastoids and subtracting the average mastoids activity from the active electrode is preferred due to its easy applicability and low impedance characteristics (Yao et al., 2005). LM also has an advantage that it can be used in low density ERP recordings as it is independent of the number of electrodes. Since, there is no electrically neutral point on the scalp or the body, referencing to mastoids may affect the characteristics of the ERPs i.e., amplitude and latencies (Yao et al., 2005, 2007) and influence the interpretability of the ERPs.

Another popular referencing procedure used widely across research laboratories is the average referencing (AVG; Offner, 1950; Nunez et al., 2001). The assumption underlying AVG is that the common average of all the recorded EEG channels will approximate zero and, hence AVG can be considered a neutral reference. This assumption is contingent on extensive spatial sampling which is achieved by using a dense electrode montages covering the full head surface for recording (Yao et al., 2005; Kayser and Tenke, 2010; Luck, 2014). However, commonly used electrode montages 32-channel, 64-channel and even the high density 128-channel electrode montages are not enough to cover the whole head surface as these montages will cover only the upper part of the head. The average activity from the channel cannot approximate zero or neutral potential inducing bias in the EEG recordings (Dien, 1998). Since, ERP laboratories across the world use various scalp-electrode montages (16, 32, 64, or 128-channel), and given the dependence of AVG on the montage, the results of various investigations may not be comparable. Nevertheless, AVG remains a popular alternative referencing procedure similar to LM in the investigation of auditory ERPs (Ponton et al., 2000; Bishop et al., 2007).

Another referencing technique which has been gaining popularity among EEG researchers is reference electrode standardization technique (REST) developed by Yao (2001). The premise behind the REST approach is the concept of infinity reference referring to a point far away from the brain sources and having an ideal neutral reference (Yao, 2001; Yao et al., 2005; Qin et al., 2010). In this approach, the recorded EEG (referenced to any scalp point) is transformed to the potentials referenced to a point located in the infinity, i.e., the neutral reference (Yao, 2001; Chella et al., 2016). The REST approach suffers from same limitations as that of average reference emitting from insufficient electrode density, head surface coverage, and inaccurate knowledge of head model (Yao, 2001; Zhai and Yao, 2004; Liu et al., 2015). However, in simulation studies REST approach has been observed to offset the disadvantages of LM and AVG referencing procedures. It recovers the spatio-temporal characteristics and the power information of the recorded EEG at the scalp level (Yao, 2001; Qin et al., 2010). There have been a few studies comparing the effects of REST technique with other commonly used referencing approaches on EEG power, functional connectivity and default mode network analysis (Yao et al., 2005; Qin et al., 2010; Chella et al., 2016). These investigations have reported that different referencing procedures result in changes in the EEG power, functional connectivity, and default network measures but all recommended using REST for referencing to offset the variability in these EEG domains.

There is limited research examining the effect of referencing procedures on ERPs. Joyce and Rossion (2005) compared AVG, LM, averaged earlobes, non-cephalic, and nose referencing sites on face-sensitive N170 and vertex positive potential (VPP). They found that while referencing procedures did not affect the latencies of these potentials, the amplitude varied drastically with reference sites for both VPP and N170. They concluded that the discrepancy of the N170/VPP effects across studies could be explained by the difference in reference methodology. Similarly, Yao et al. (2007) compared unilateral mastoids, LM, AVG, vertex reference, and the infinity reference (REST) for temporal and spatial characteristics of four peaks of somatosensory evoked potentials (P30, P40, N90, and P230). The results revealed that referencing procedures influenced the amplitude of all the four peaks across many electrodes but did not change the relative scalp distribution of the potentials. They recommended the use of a common referencing approach across the laboratories. In an another study, Tian and Yao (2013) investigated the influence of average reference, linked mastoids and infinity reference on experimental effects on ERPs elicited using audiovisual stimuli. They found that the scalp distribution of N1 potential was similar (posterior) when REST referenced validated by two spatial analyses methods (SPSM and LORETA). AVG and LM referencing produced different results with these methods. It was suggested that using REST in ERP analyses will increase the accuracy of ERP results. Recently, Chella et al. (2017) reported effectiveness of REST procedures for the analyses of non-linear features of EEG such as frequency analyses for both simulation and real EEG experiments for a less dense 21-channel montage as well. Substantial evidence exist comparing different referencing procedures including REST, LM, and AVG in simulation studies (Liu et al., 2015; Chella et al., 2016, 2017), functional connectivity analyses (Qin et al., 2010), source analyses (Tian and Yao, 2013), with results proving the validity and effectiveness of REST in these EEG measures. Following this promising evidence, a comparative assessment of commonly used referencing procedures such as LM and AVG needs to be conducted against REST to extend the validity of the infinity referencing procedures in the ERP domain as well.

To our knowledge there is no report that has examined the effect of different referencing techniques on auditory ERPs. It is essential to compare different referencing procedures because research on auditory processing using auditory ERPs in basic and clinical audiological and psychological research has used myriad referencing procedures across the research laboratories. Using different referencing procedures for a same research question can result in different inferences and cross-study comparisons across research laboratories may not be comparable. In these cases, it would be constructive to know the effect different referencing procedures (commonly used) may have on auditory ERPs to facilitate uniform comparisons and using a common referencing procedure across the research and clinical laboratories.

In the current experiment, the influence of three commonly used referencing approaches, LM, AVG, and REST was measured on amplitude and latencies of the mismatch negativity (MMN), the auditory ERP. The MMN is a low level cognitive potential and is thought to represent pre-attentive sensory memory (Näätänen, 1992), auditory discrimination (Naatanen et al., 2005; Sharma et al., 2006; Mahajan and McArthur, 2015), and redirection of focussed attention (Naatanen et al., 2005). The auditory MMN can be measured by subtracting an ERP elicited by a frequently occuring “standard” sound from an ERP elicited by an infrequent “deviant” sound of certain physical attribute (frequency, duration, or intensity). In adults, the MMN typically presents as a negative response found 100–250 ms after the onset of a sound. It is established that large frequency differences between standard and the deviant sounds elicit large amplitude and shorter latency of MMN, where as a small difference results in smaller and prolonged latencies of MMN (Tiitinen et al., 1994; Novitski et al., 2004; Kujala et al., 2007). This experimental effect was investigated in the current study. Following Tian and Yao (2013), we investigated the effect of referencing on the experimental effect (increasing difference in deviant and standard frequency) rather than the absolute auditory ERPs. The effect of referencing will be more pronounced on the experimental effects than the absolute ERPs (Tian and Yao, 2013) resulting in varied inferences about the investigated research questions depending upon the reference used.

Given that there are no direct comparisons on the effect of referencing procedures on auditory MMN and the importance of MMN in clinical and basic auditory processing research, the aim of the current experiment was to examine how the choice of ERP referencing procedure will influence the latency and amplitude of the MMN as a function of the magnitude of frequency deviance between the standard and the deviant sounds. Also, given the dependence of AVG and REST referencing procedures on the electrode montage we examined the influence of AVG and REST referencing on the MMN amplitude and latency as function of three commonly used electrode montages (16-, 32-, and 64-channel). Less dense montages are usually recommended for recording auditory MMN for clinical use (Duncan et al., 2009). Since, the LM and AVG are considered non-neutral references as compare to REST, it would be reasonable to predict that an increment in MMN amplitude and decrement in MMN latency as a function of increasing deviant frequency magnitude will be better exhibited in the REST condition.

## Method

### Ethics

Methods were approved by the Human Research Ethics Committee at the Western Sydney University. Written informed consents were obtained from all the participants prior to the experiment.

### Participants

Twelve participants (7 females), aged 22–35 years participated in the experiment. All the participants were right handed as assessed by Edinburgh handedness inventory and reported no significant neurological and psychological history. Routine hearing screening audiometry conducted revealed normal hearing bilaterally with hearing thresholds of ≤ 15 dB HL at 500, 1,000, and 2,000 Hz for all the participants.

### Experimental stimuli

The experimental stimuli consisted of four pure tones (175, 10 ms rise and fall time) with the frequency of, 1,000, 1,030, 1,100, and 1,200 Hz. The 1,000 Hz pure tone served as the “standard stimulus” in all three blocks and was presented at 85% of the trials. In each block the standard stimulus were replaced in 15% of the trials either by 1,030, 1,100, or 1,200 Hz pure tone termed the “small-deviant,” “medium-deviant,” and “large-deviant.” Each block contained 666 stimuli (566 standards; 100 deviants) that were presented binaurally via headphones at 80 dB SPL. Each block started by 10 repetitions of the standard stimulus after which the standards and deviants were presented in a pseudo-random order with the constraint that a minimum of three standards and a maximum of eight standards were presented between the deviants. The stimuli were separated by a jittered stimulus-onset synchrony (SOA) of 0.7–0.9 s to minimize the confounding effect of ERP artifacts related to anticipation of a stimulus and overt attention (P3a and P3b; Lang et al., 1995). An increasing magnitude in frequency was used as deviants, as MMN generated by increase in frequency in the deviant stimulus produce larger amplitude than MMN generated by decrease in frequency of deviant (Peter et al., 2010). The participants were instructed to ignore the sounds and they watched a subtitled muted movie of their choice to divert their attention. The soundtrack of the movies was switched off to avoid any inhibitory effects on the MMN component (Pettigrew et al., 2004; Mahajan and McArthur, 2011). The order of the blocks was counter-balanced between the participants.

### Electrophysiological recording

The participants were seated on a comfortable chair while the electrode cap was fitted. Prior to the electrode cap placement, the scalp of each participant was combed in a pre-set procedure to reduce the time taken to achieve the optimal scalp electrode impedance (Mahajan and McArthur, 2010). A BioSemi Active-Two amplifier system (BioSemi, Amsterdam, Netherlands) was used to record raw electroencephalograph (EEG). The 64 Ag-AgCl electrodes were mounted on a nylon electrode cap according to the international standard 10–10 system (Oostenveld and Praamstra, 2001). There were two electrodes on the electrode cap (CMS and DRL) which served as online references. Six additional electrodes were also placed on the participants. Four of them were bipolar electrodes placed above and below the left eye and outer canthi of both the eyes to monitor vertical and horizontal eye movements (EOG channel) respectively and two electrodes were placed on two mastoids which were used for re-referencing later. The raw EEG recording was sampled at 256 Hz with online band-pass filtering of 0.05–200 Hz. This raw EEG data was stored for later offline analysis for each participant.

### EEG data analysis

**Figure 1:**
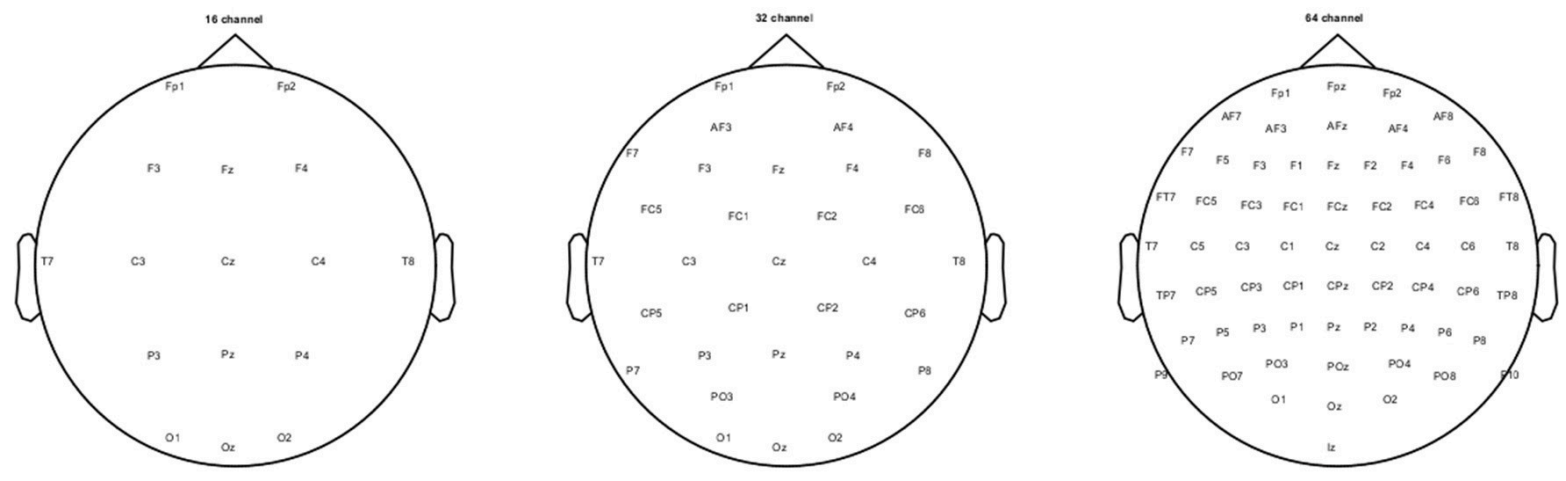
16-, 32-, and 64-channel electrode montages used in the current study.

The pre-processing and analysis of the stored raw EEG data from each participant was carried out using EEGLAB version 13.2 (Delorme and Makeig, 2004), ERPLAB toolbox version 5.0 (Lopez-Calderon and Luck, 2014) and custom written functions in MATLAB 2014b (Mathworks, Natick, MA, USA). Initially, any obvious artifact was removed after visually inspecting the continuous raw data. Then this data was band-pass filtered (0.1 Hz high pass and 30 Hz low pass; 12 dB per octave roll-off) using finite FIR filters. The filtered data then was subjected to *runica*, an ICA (Independent component analysis) algorithm incorporated in EEGLAB to detect and remove eye blinks, horizontal eye movements, and other artifacts (muscle noise and line noise artifacts). The ICA algorithm resulted in 64 components and based on the scalp topography, activity power spectrum, and activity over trials, the artifactual components were identified and removed from the EEG data. The ICA-corrected resultant EEG activity was then divided into three scalp-electrode montages namely, 64-, 32-, and 16-channel, according to the international standard 10–10 system (see Figure 1). At this stage of the data processing, a three-way re-referencing of the continuous data was performed to create three different experimental referencing conditions for each electrode montage separately.

In the first method, the ICA-corrected data was re-referenced to the average of two mastoids that were mathematically linked for each of the three electrode montages and formed the first referencing condition termed as “linked-mastoid referencing” (LM). For the second method, the data was averaged referenced to all the channels with in each electrode montage i.e., averaged reference to 64-, 32-, and 16-channel. This condition was termed as “AVG.” Third, the ICA-corrected data in each electrode montage was subjected to REST referencing procedure using the REST EEGLAB plugin resulting “REST referencing.” A three-concentric-sphere model was used as the head model for REST. The radii of the three concentric spheres are 0.87 (inner radius of the skull), 0.92 (outer radius of the skull) and 1.0 (radius of the head), and the conductivities are 1.0 (brain and scalp) and 0.0125 (skull). The lead field matrix was calculated for all the montages separately by the forward theory proposed in Yao (2000), following which the data was referenced to the REST.

The resulting re-referenced data in each of the referencing condition and electrode montage was divided into 800 ms epochs with a pre-stimulus period of 100 ms, which was used for the baseline correction. All the epochs with a voltage change exceeding ± 100 μV in any channel were removed from the analyses. All the participants had at least 80 accepted trials for each deviant. All epochs generated for each referencing condition and electrode montage by the 1,000 Hz standard tone were averaged (excluding the first 10 standards and the standards that immediately follow a deviant) together to produce a “standard” ERP. All epochs generated by 1,030, 1,100, and 1,200 Hz tones were averaged to produce the “small,” “medium,” and “large” deviant ERPs, respectively. To create the MMN, the standard ERP were subtracted from each of the small, medium, and large deviant ERP (i.e., a difference waveform) which produced three difference waveforms for each referencing condition.

The MMN was identified from the difference waveforms for each condition and each electrode montage. We focused on the frontal channel Fz to measure the amplitude and the latency of the MMN, as Fz is the site most commonly used to represent the MMN (Näätänen, 1992; Jacobsen and Schröger, 2003; Naatanen et al., 2005; Kujala et al., 2007). Also at Fz, comparable auditory ERPs and reliable group differences can be found (Ponton et al., 2000; Naatanen et al., 2005; Bishop et al., 2007; Mahajan and McArthur, 2012, 2015). The largest negative deflection between 100 and 250 ms was identified as MMN for each condition and MMN mean latency was measured at this point. The MMN amplitude was indexed as the mean amplitude of the MMN waveform over a 50 ms window (Peter et al., 2010, 2012; Mahajan and McArthur, 2011, 2015), centered on the peak latency for each participant.

### Statistical analyses

The MMN amplitude and latency measurements (54 datasets) were subjected to the Shapiro-Wilk test of normality. The results revealed that the MMN amplitude and latency data across the electrode montages and referencing procedures were normally distributed (*p* < 0.05). To determine the effect of referencing procedures on the magnitude of auditory MMN as a function of deviance magnitude across three electrode montages, the data on the mean amplitude and peak latency was subjected to two separate analyses. (1) To see the effect of referencing procedure on MMN amplitude and latency across the deviant magnitudes, a 3 (“deviant magnitude”; small, medium, and large) × 3(“referencing procedure”; LM, AVG, and REST) within participant repeated measures analysis of variance (ANOVA) was conducted for each electrode montage separately. (2) To see the effect of referencing procedure, electrode montage, and the deviance magnitude on MMN, a 2 (“referencing procedure”; average reference and REST) × 3 (“montage”; 16-, 32-, and 64-channel) × 3 (“deviant magnitude”; small, medium, and large) was performed. LM referencing was not included in this analysis as MMN at Fz for LM condition is independent of the electrode montage. For all the above ANOVA analyses, wherever the assumption of sphericity was violated the Greenhouse-Geisser correction was applied. The results obtained are reported in the section below.

## Results

**Figure 2:**
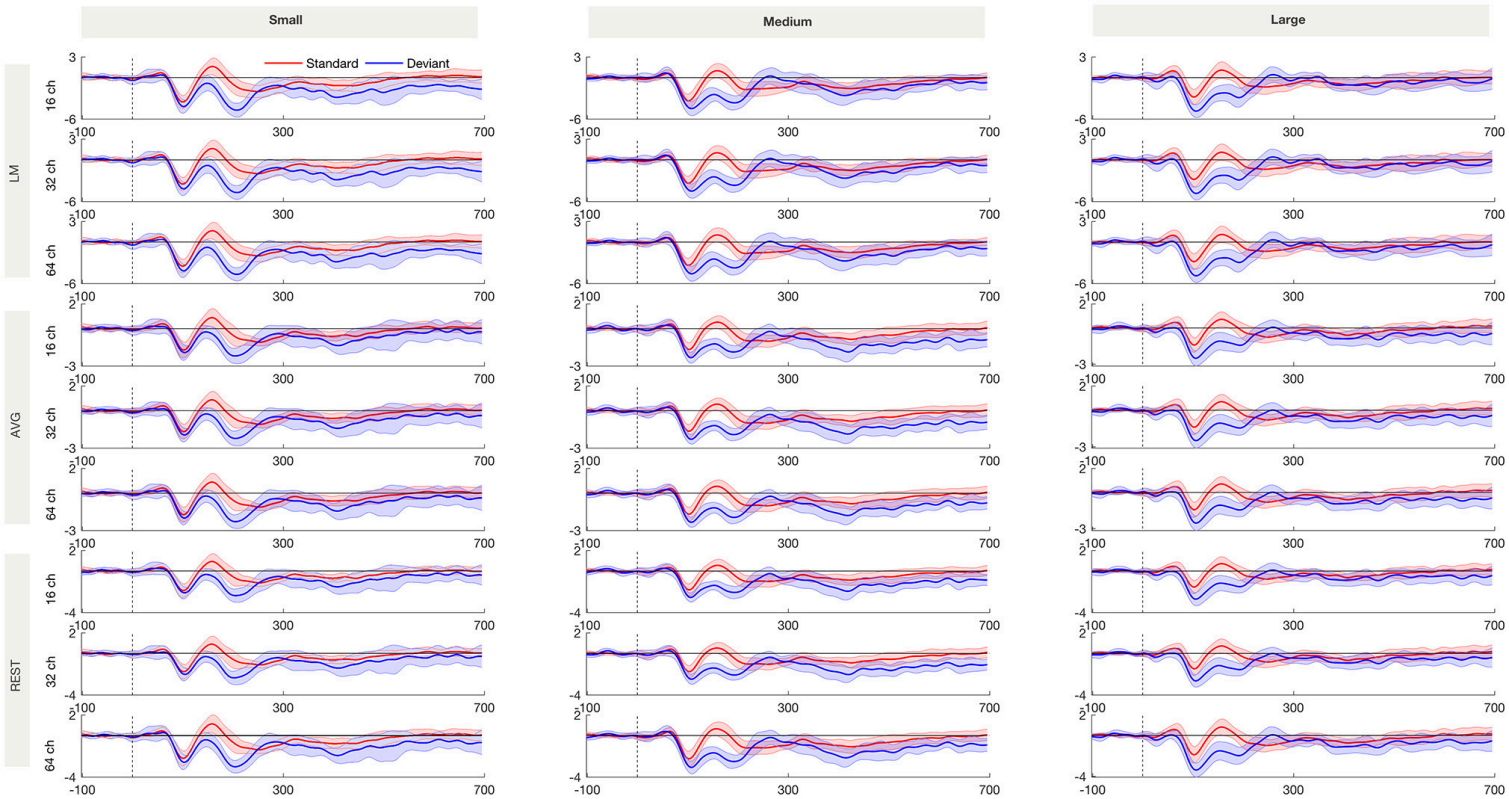
Standard and deviant waveforms referenced with LM, AVG, and REST procedures recorded from Fz for small, medium and large frequency deviance across 16, 32, and 64-channel montages. The shading encompasses 95% Cousineau- Morey confidence intervals (Morey, 2008).

**Figure 3:**
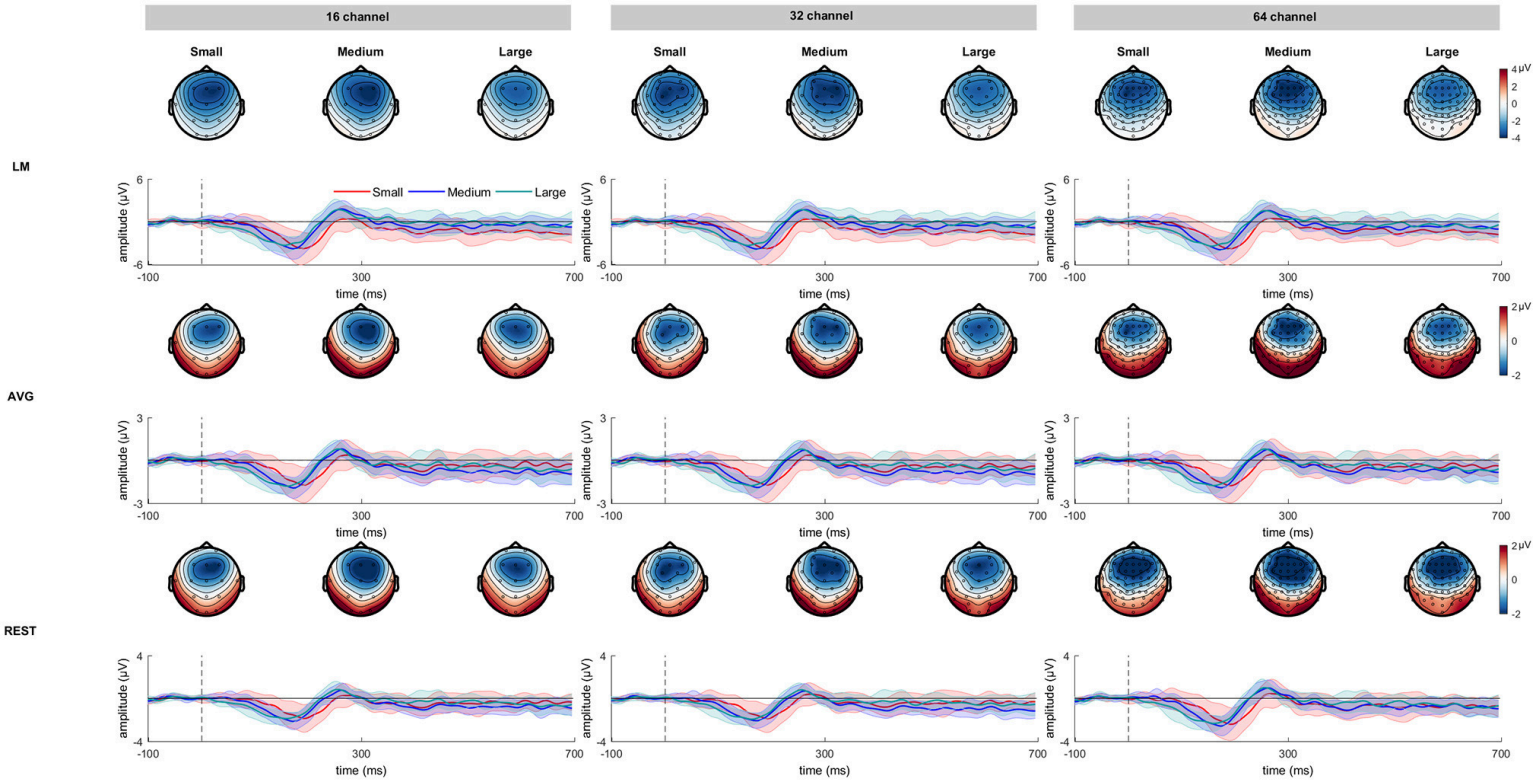
The deviant minus standard difference waveforms as a function of frequency deviance across three channel montages referenced at LM, AVG, and REST procedures. The corresponding topographical maps for small, medium, and large frequency deviance representing a strong fronto-central distribution are also shown. The shading encompasses 95% Cousineau- Morey confidence intervals (Morey, 2008).

Figure 2 shows the grand averaged standard and deviant ERP waveforms re-referenced using LM, AVG and REST procedures across 16-, 32- and 64-channel montages. Figure 3 shows the grand average deviant-standard difference waveforms across different referencing procedures and montages. The topographic maps show the distribution of MMN amplitude at its peak across the scalp.

### Amplitude of MMN

The results of the 2-way repeated measures of ANOVA with factors magnitude (small, medium, large) and referencing procedure (LM, AVG, REST) revealed that there was no main effect of magnitude of deviance on MMN amplitude for all the montages [64-channel; *F*_(2, 22)_ = 0.09, *p* = 0.91, $\eta_{p}^{2}$ = 0.008; 32-channel *F*_(2, 22)_ = 0.09, *p* = 0.91, $\eta_{p}^{2}$ = 0.008; 16-channel *F*_(2, 22)_ = 0.09, *p* = 0.91, $\eta_{p}^{2}$ = 0.008], However, there was a significant main effect of the referencing procedure for all the montages [64 channel *F*_(2, 22)_ = 88.61, *p* < 0.001, $\eta_{p}^{2}$ = 0.90; 32-channel *F*_(2, 22)_ = 94.90, *p* < 0.001, $\eta_{p}^{2}$ = 0.90; a16-channel *F*_(2, 22)_ = 93.58, *p* < 0.001, $\eta_{p}^{2}$ = 0.90]. These results indicate that for all the three electrode montages, the change in the magnitude of frequency deviance did not alter the mean MMN amplitude across the three referencing procedures but the choice of referencing procedure influenced the MMN amplitude significantly. Bonferroni corrected pairwise comparisons revealed that, for 64-channel montage when the EEG data was re-referenced to mastoids, largest MMN amplitude was obtained (M = –3.39 μV, SE = 0.41) followed by the REST referencing (M = −2.25 μV, SE = 0.31) which was significantly larger than the AVG (M = −1.67, SE = 0.26). Similar results were obtained for MMN amplitude for 32-channel, LM (M = −3.36 μV, SE = 0.40), REST (M = −1.80 μV, SE = 0.29) AVG (M = −1.66 μV, SE = 0.25) and 16-channel LM (M = −3.34 μV, SE = 0.40), REST (M = −1.80 μV, SE = 0.26) AVG (M = −1.67 μV, SE = 0.26). There were no significant interactions between the variables “referencing procedures” and “magnitude of deviance” for all the electrode montages. It is expected that the amplitude of MMN at Fz should be identical for all the three montages for LM. The small change in MMN amplitude across the montages in the present study could be due to the small difference in the number of artifact free trials across montages. Since the artifact rejection (± 100 μV criterion) was applied on all the channels in the montage, there are slight differences in the number of artifact free trials across 16-, 32-, and 64-channel montages. This made the ERPs slightly different across montages.

The three-way ANOVAs with factors magnitude (small, medium, large), montage (16-, 32-, 64- channel) and referencing procedure (AVG, REST) revealed that while there was no main effect of magnitude of deviance on MMN amplitude [*F*_(2, 22)_ = 0.14, *p* = 0.91, $\eta_{p}^{2}$ = 0.01], there were significant main effects of electrode montage [*F*_(2, 22)_ = 30.86, *p* < 0.001, $\eta_{p}^{2}$ = 0.73], and referencing procedures [*F*_(2, 22)_ = 53.11, *p* < 0.001, $\eta_{p}^{2}$ = 0.83] on the mean amplitude of MMN. These results suggest that AVG and REST procedures influenced the MMN amplitude across the electrode montages and change in deviance did not alter the MMN amplitude across these two procedures and montages. Among the three different scalp electrode montages across both these referencing procedures, Bonferroni corrected pairwise comparisons showed that the largest MMN was obtained when 64-channel were used (M = −1.96 μV, SE = 0.28) as compared to 32-channel (M = −1.73 μV, SE = 0.27) and 16-channel montage (M = −1.73 μV, SE = 0.26). Similar to previous findings MMN generated through REST referencing elicited larger amplitude (M = −1.95 μV, SE = 0.28) than the AVG referencing (M = −1.67 μV, SE = 0.26) across all the montages. “Referencing procedures” and “montage” significantly interacted with each other [*F*_(2, 22)_ = 40.72, *p* < 0.001, $\eta_{p}^{2}$ = 0.79]. There were no other significant interactions between the independent variables.

To investigate the significant interaction between referencing procedure and montage, subsequent one-way ANOVAs were conducted for each referencing procedure separately. The results revealed that when the EEG data was re-referenced using REST procedure, the mean MMN amplitude was largest for 64-channel montage (M = −2.25 μV, SE = 0.31; *F*_(2, 10)_ = 25.09, *p* < 0.001, $\eta_{p}^{2}$ = 0.83] which was significantly different from MMN amplitude using 32-channel montage (M = −1.79 μV, SE = 0.29) and 16-channel montage (M = −1.80 μV, SE = 0.27). There was no difference in mean MMN amplitude between 32 and 16-channel montages when re-referenced using REST. Also, the mean MMN amplitude did not differ across the montages when the EEG data was referenced to AVG procedure [*F*_(2, 10)_ = 0.20, *p* = 0.81, $\eta_{p}^{2}$ = 0.03].

In summary, the magnitude of deviance did not have an effect on MMN amplitude. LM had largest MMN amplitude followed by REST and AVG referencing had MMN with smallest amplitude. REST referencing depended on the montage with 64-channel montage generating larger MMN response compared to 32 and 16 channel.

### Latency of MMN

The results of two way ANOVAs revealed that for 64-channel there was a significant main effect of magnitude of frequency deviance on the latency of MMN [*F*_(2, 22)_ = 4.99, *p* = 0.01, $\eta_{p}^{2}$ = 0.31]. Bonferroni corrected pairwise comparisons revealed that large difference between standards and deviants (1,200 Hz; M = 153 ms, SE = 5.28) had significantly shorter latency of MMN than medium difference (M = 175 ms, SE = 7.44) or small difference (M = 184 ms, SE = 7.09). No difference was found between medium and small magnitude of frequency deviance. The referencing procedures did not have any main effects on the latency of MMN [*F*_(2, 22)_ = 0.13, *p* = 0.87, $\eta_{p}^{2}$ = 0.01]. Similar significant main effect of magnitude of frequency deviance was found on MMN latency for both 32-channel [*F*_(2, 22)_ = 4.96, *p* = 0.01, $\eta_{p}^{2}$ = 0.31] and 16-channel [*F*_(2, 22)_ = 6.22, *p* = 0.007, $\eta_{p}^{2}$ = 0.36] scalp electrode montages. For these electrode montages as well, large deviance elicited the shortest MMN latency (M = 154 ms, SE = 5.45, 32-channel; M = 153 ms, SE = 5.29, 16-channel) than medium (M = 172 ms, SE = 7.47, 32-channel; M = 174 ms, SE = 7.37, 16-channel) and small deviances (M = 187 ms, SE = 7.88, 32-channel; M = 185 ms, SE = 6.66, 16-channel). There was no main effect of referencing procedures for these channel montages as well and none of these independent variables significantly interacted with each other.

The results of the three way repeated measures of ANOVA analyses also revealed a significant main effect of the magnitude of frequency deviance across three electrode montages and sAVG and REST referencing procedures [*F*_(2, 22)_ = 5.39, *p* = 0.01, $\eta_{p}^{2}$ = 0.33]. This suggests that increasing the frequency deviance shortened the MMN latency for both the referencing procedures and all the three scalp-electrode montages (M = 151 ms, SE = 6.96, large; M = 174 ms, SE = 7.29, medium; M = 185 ms, SE = 7.56, small). The MMN latency for large deviance significantly differed from medium and small deviance, with no difference between the latter two.

## Discussion

The primary aim of the current study was to determine the extent to which choice of referencing procedure influence the amplitude and latency of the auditory ERP, the MMN as a function of magnitude of frequency deviance across three different electrode montages. To this end, we compared the effects of LM, AVG, and REST referencing procedure on the MMN as a function of increase in deviance magnitude and 64, 32, and 16-channel scalp-electrode montages. The results revealed that the referencing procedure did not alter the MMN amplitude and latency as a function of frequency deviance across the three montages. While the magnitude of frequency deviance did not change the amplitude of the MMN, the latency of the MMN decreased with increase in the frequency deviance. For all the three scalp-electrode montages, the LM referencing procedure elicited the largest MMN amplitude across the magnitude of deviance. After applying REST referencing on the EEG data, the MMN amplitude was found to be largest for the EEG data recorded from 64-channel than other two electrode montages.

The experimental effect used in the current study (increasing the frequency deviance) was validated with the findings of reduction in the latency of the MMN with an increase in the difference between the standard and the deviant frequencies used in oddball presentation. This finding is in agreement with previous results (Sams et al., 1985; Tiitinen et al., 1994; Novitski et al., 2004; Kujala et al., 2007). These results reinforce the established fact that the MMN accurately reflects auditory discrimination ability and the speed of neural conduction underlying auditory discrimination is faster when the difference between the standards and the deviants is large. No effect of an increment of the frequency deviance was found on the amplitude of the MMN. This result was contrary to the common prediction of large amplitude with increase in the frequecy difference between the sounds (Kujala et al., 2007) but in agreement with a previous finding (Horvath et al., 2008). Horvath et al. (2008) suggested that the MMN amplitude reflects the neural representation of percentage of deviants detected rather than the neural representation of the index of magnitude of deviance. This means that if the difference between the standards and deviants is large enough (greater than the threhsold frequency discrimination level), index of deviance detection will be high irrespective of the deviance difference resulting in no change in the amplitude of MMN across deviance.

Although the MMN latency changes as a function of magnitude of frequency deviance, there was no effect of three referencing procedures (LM, AVG, and REST) on this experimental effect. This result suggests that the experimental effect of change in MMN latency as a function of frequency deviance is not contingnent on choice of referencing procedure for the three commonly used electrode montages. These results are not in agreement with previous research that investigated effect of referencing procedures on the experimental effects in sensory perception (Joyce and Rossion, 2005; Tian and Yao, 2013) and found that choice of referencing alters the evoked potentials. Lower electrode density, incomplete head coverage and inaccurate head model are some known disadvantages of the AVG and REST referencing procedures (Yao, 2001; Zhai and Yao, 2004; Liu et al., 2015). These limitations can results in contamination of the resultant electrical potential, (auditory MMN in this case) with non-zero potentials. It is possible that due to these limitations, there was no effect of AVG or REST procedures on the desired experimental changes in MMN amplitude and latencies. It should be noted however, that recent simulation and real EEG studies have reported the effectiveness of REST for the analysis of non-linear features of EEG data for montages with 21 channels (Chella et al., 2017). To our knowledge, this is the first empirical investigation of comparing the effects of three commonly used referencing procedures on experimental effects on auditory MMN. Further research is required to identify the effect of referencing procedure on the experimental manipulations using other auditory contrasts (duration, speech discrimination etc.), more complex paradigms (abstract MMN) and denser electrode montages such as 128 and 256 channels.

The results of the current study revealed that when the EEG data was re-referenced to the linked mastoids, the MMN amplitude was the largest for all three scalp-electrode montages used. LM referencing has been recommended when the amplitude of MMN is small (Picton et al., 2000; Kujala et al., 2007). For frequency deviance, enhanced MMN is reported at Fz scalp location due to the source orientation of the MMN generators (Deouell et al., 1998; Kujala et al., 2007). In the LM referencing, the distance from the reference sites is equidistant to Fz leading to a strong representation of the underlying MMN generating dipole at Fz, which will result in enhanced MMN amplitude. The synchronous activity of the neurons underlying the generation of MMN is coordinated by a neural dipole which has a negative and a positive end. The LM referencing enables addition of negative and positive components of the MMN response (dipole), resulting in a higher signal-to-noise ratio (SNR) of the MMN and hence a larger amplitude.

Recent research in search of a gold standard referencing procedure for EEG/ERP research have determined that the potential at mastoid electrode sites used for referencing is non-zero (Yao, 2001, 2017; Chella et al., 2017). When the EEG data is re-referenced to LM, the non-zero potentials at mastoids may get added or subtracted to the potential at the active electrode in effect increasing or decreasing its amplitude. In the current study, the non-zero components may have got added up to the true potential resulting in larger MMN amplitude. This result may also suggest that when sparse electrode montages are used (e.g., 16-channel) such as in case of young children or clinical participants, linked mastoid referencing may be used that will elicit large MMN amplitude and can facilitate experimental comparisons. However, at the same time caution must be exercised while interpreting these results as the resultant ERP may constitute both true experimental and non-zero potential from the mastoids.

The amplitude of ERPs generated from AVG and REST referencing procedures, is generally found to be smaller than LM reference. The potential calculated using REST and AVG referencing can be non-zero in case of less electrode density, non-whole brain coverage and non-spherical head shape (Chella et al., 2017; Yao, 2017). In such cases, the non-zero component from the channels contributes to the resultant potential at active electrode sites. The scalp electrode montages employed in the current study also did not cover the whole scalp and it is plausible that the non-zero potentials across the channels were subtracted from true potential leading to a reduction in the MMN amplitude for AVG and REST referencing procedures across the three channel montages compared to LM reference. Given that we used a three-layered spherical model for the REST approach, the EEG data referenced with REST may show a better MMN reconstruction than AVE referencing for low density electrode montages such as used in the current study (Chella et al., 2017). This could be a possible explanation for larger MMN amplitude when computed after REST referencing than AVG referencing.

Another crucial set of results obtained from the current study revealed the level of dependence of two reference-free procedures (AVG and REST) on the electrode montages. The MMN amplitude when computed using AVG referencing was not contingent on the number of electrodes of the electrode montage with no difference in MMN amplitude across the three montages. This result is contrary to the common consensus that denser the electrode array better will be the effectiveness of the AVG referencing to have a neutral potential that would result in reconstruction of the true potential from an electrode site. The MMN amplitude resulting from application of AVG referencing was the smallest across three electrode montages when compared to LM or REST referencing. The smaller size of the MMN here might have impeded any interaction between the AVG referencing and the electrode montages. Also, recent EEG simulation experiment have shown that electrode density may not be a critical factor when using AVG referencing as compared to the montage coverage of the scalp (Chella et al., 2016, 2017; Yao, 2017). The non-dependence of AVG referencing on montage density may have led to no differences in MMN amplitude across 16, 32, and 64-channel montages in the current study.

On the other hand, when infinity reference technique i.e., REST was applied to compute MMN amplitude, it was revealed that, the size of MMN amplitude is dependent on the number of electrodes used in the reconstruction of the potential. The MMN amplitude was the greatest when REST procedure was applied on a 64-channel montage as compared less dense arrays of 32 and 16-channel. This result is related to the previous findings of simulation studies using REST that reported, denser the electrodes (in a montage) better the reconstruction of the target potential (Yao, 2001; Zhai and Yao, 2004). The results of these simulation studies established that when the electrode montage had more than 32 electrodes, the quality of the reconstructed potential was better than when constructed from AVG and LM referencing. Larger MMN amplitude computed with REST referencing with 64-channel montage when compared to AVG referencing suggests that REST referencing is more dependent on the denser electrode montage used for recording auditory ERPs than AVG referencing.

## Conclusion

The results of the current study indicate that (1) experimental effect of magnitude of frequency deviance on MMN amplitude and latency do not depend on the choice of referencing procedure (LM, AVG, or REST). (2) Auditory MMN will be largest if the EEG data is referenced with LM followed by REST and then AVG referencing. (3) MMN amplitude computed using REST referencing depends on the number of electrodes used in the montage with 64-channel montage producing largest MMN amplitude. (4) The MMN amplitude elicited using average AVG referencing did not depend on the electrode montage.

The results of the present study contribute to the increasing empirical investigations regarding the use of infinity referencing procedures in various electrophysiological domains. This is the first study investigating the effects of two commonly used referencing procedures (LM and AVG) and REST referencing on auditory ERP the MMN as a function of frequency deviance. While the results revealed no effect of referencing procedures on auditory MMN amplitude and latency as a function of frequency deviance, it is possible that the AVG and REST may have suffered from limited scalp electrode coverage and the sparse scalp electrode density in the current study. Though the utility of REST in less dense montages have been proved recently, there is a probability that 16, 32, and 64-channel montages used in the current study might have altered the final MMN waveforms across the experimental conditions. Hence, to confirm and extend these findings, replication studies using dense montages such as 128 and 256-channels and different experimental manipulations such as change in duration deviance to elicit MMN should be employed in future.

## Author contributions

YM, VP, and MS designed the study. YM conducted the experiment. YM and VP analyzed and interpreted the data. YM, VP, and MS wrote the paper. All authors approved the final version of the manuscript.

### Conflict of interest statement

The authors declare that the research was conducted in the absence of any commercial or financial relationships that could be construed as a potential conflict of interest.
